# Pattern of renal amyloidosis in South Africa

**DOI:** 10.1186/s12882-019-1601-x

**Published:** 2019-11-09

**Authors:** Muhammed Hassen, William Bates, Mohammed Rafique Moosa

**Affiliations:** 1 Division of Nephrology, Stellenbosch University and Tygerberg Hospital, Cape Town, South Africa; 2 Department of Anatomical Pathology, Stellenbosch University and Tygerberg Hospital, Cape Town, South Africa

**Keywords:** Amyloidosis, HIV, Tuberculosis, Chronic kidney disease

## Abstract

**Background:**

Kidney disease is a serious manifestation of systemic amyloidosis and a major cause of morbidity and mortality. Tuberculosis (TB) occurs up to 27 times more commonly in human immunodeficiency virus (HIV) infected patients and is also an important cause of renal amyloid; there are however no reports of renal amyloidosis in South Africa in the HIV era.

**Methods:**

This was a retrospective record review of cases of amyloidosis diagnosed on renal biopsies at our tertiary referral hospital between January 1985 and December 2016.

**Results:**

Forty-six cases of amyloidosis were identified over the study period. The calculated biopsy prevalence was 1.38 per 100 non-transplant renal biopsies (95% Confidence Interval 1.02–1.86). AL amyloidosis was identified in 26 (57%) cases and AA in 20 (43%). The median age at presentation was 51 years and 52% of cases were female. Patients with AA amyloidosis were significantly younger compared to their AL counterparts (age 42 years vs. 58 years, *p* = < 0.001) and were all significantly non-white. The main clinical presentation was nephrotic syndrome (85%) and 52% of cases also had a serum creatinine value of greater than 120 μmol/L. Of the 20 cases of AA amyloidosis, 12 (60%) were associated with tuberculosis. HIV infection was noted in only two (10%) of the 20 AA cases. Median survival after diagnosis was 2 months.

**Conclusion:**

Amyloidosis is a rare cause of kidney disease and typically presents with nephrotic syndrome. A similar number of AA and AL types were observed, and outcomes are worse in cases of AA amyloid. While TB remains the major underlying disease in this type, HIV infection was infrequent in cases of AA renal amyloidosis.

## Background

Renal manifestation of systemic amyloidosis carries a significant morbidity and mortality, and is most frequently seen either in the setting of chronic inflammation (AA amyloidosis), or plasma cell dyscrasias (AL amyloidosis) [1–5]. Without treatment, amyloidosis associated kidney disease progresses rapidly to end-stage renal disease (ESRD). A recent study reported dialysis dependence within 18 months of diagnosis of AA amyloidosis and mean patient survival of 52.9 months, whilst these outcomes were 36.3 months and 50 months respectively in AL amyloid [4, 6].

Marked differences in the epidemiological patterns of amyloidosis are seen between different countries, highlighting the importance of local data [2, 7–9]. AL amyloidosis is more prevalent in developed countries, whilst AA amyloidosis is more common in the developing world [2, 7, 8, 10]. The developmental status of a particular country, its prevalence of infectious diseases, as well as public health measures implemented to control chronic infections are dynamic factors influencing the prevalence of secondary (AA) amyloidosis over time [5, 11, 12]. Tuberculosis (TB) is a common underlying disease in AA amyloidosis, particularly in developing countries [2, 7, 9]. Underlying human immunodeficiency virus (HIV) infection increases the risk of TB 6 to 27 times compared to those that are not infected [13]. South Africa has amongst the highest rates of both TB and HIV infection in the world; the incident rate of TB was 781 cases per 100,000 population in 2016 and the estimated HIV prevalence rate was 12.57% of the total population [13, 14]. The immunocompromised state rendered by HIV increases the risk of TB disease substantially, with a TB/HIV co-infection rate in South Africa of 70% [15].

Amyloidosis is generally an uncommon histological finding on renal biopsies of HIV infected patients. Gerntholz et al. [16] identified no cases of amyloidosis among 99 patients and Wearne and Okpechi [17] a single one among 221 South African patients; Wyatt et al. [18] reported one case of amyloidosis among 89 American and as did Nebuloni et al. [19] in 73 Italian subjects. The cases of amyloidosis and HIV infection that have been reported are predominantly in intravenous drug users (IVDUs) [20–22]. Data from the pre-HIV era in South Africa suggest that AA amyloidosis is the most common type of amyloidosis seen and that TB infection is the most frequently associated risk factor [2].

We reviewed all the cases of renal amyloidosis at our institution in the context of the HIV epidemic currently prevailing in South Africa.

## Methods

### Study design

We conducted a retrospective review of consecutive cases of renal amyloidosis.

### Setting and study population

Renal biopsies performed at Tygerberg Academic Hospital between 01 January 1985 and 31 December 2016 were identified using the kidney biopsy register. This register served as the sampling frame and cases of definitive or possible amyloidosis were extracted from this source. Tygerberg Academic Hospital is a tertiary referral hospital that serves about one-half of the population of Western Cape, which was estimated at six and a half million in 2017 [14].

### Participants

All participants aged 13 years and older at renal biopsy, who had a histological diagnosis of renal amyloidosis, as defined by renal biopsy with either apple green birefringence on Congo Red stain and/or amyloid fibrils detected on electron microscopy, were included.

### Sampling technique

Records from the renal biopsy register were reviewed over the 32-year period. All cases diagnosed as definite or possible amyloidosis were evaluated. All renal pathology has been reviewed by a single experienced renal pathologist (WDB). All renal tissue was collected by percutaneous biopsy and evaluated by light microscopy, immunofluorescence and electron microscopy. Renal biopsy material was received fresh and unfixed, evaluated and divided under a dissecting microscope to provide optimal numbers of glomeruli for light and electron microscopy as well as immunofluorescent studies. The ultrastructural material was then fixed in 2.5% glutaraldehyde in 0.1 M phosphate buffer, while the light microscopy material was fixed in buffered formalin. Material for immunofluorescence was frozen, cryostat sections cut and labeled for IgA, IgG, IgM and C3. Electron microscopy (EM) specimens were post-fixed in osmium tetroxide embedded in Spurr’s resin.

**Fig. 1:**
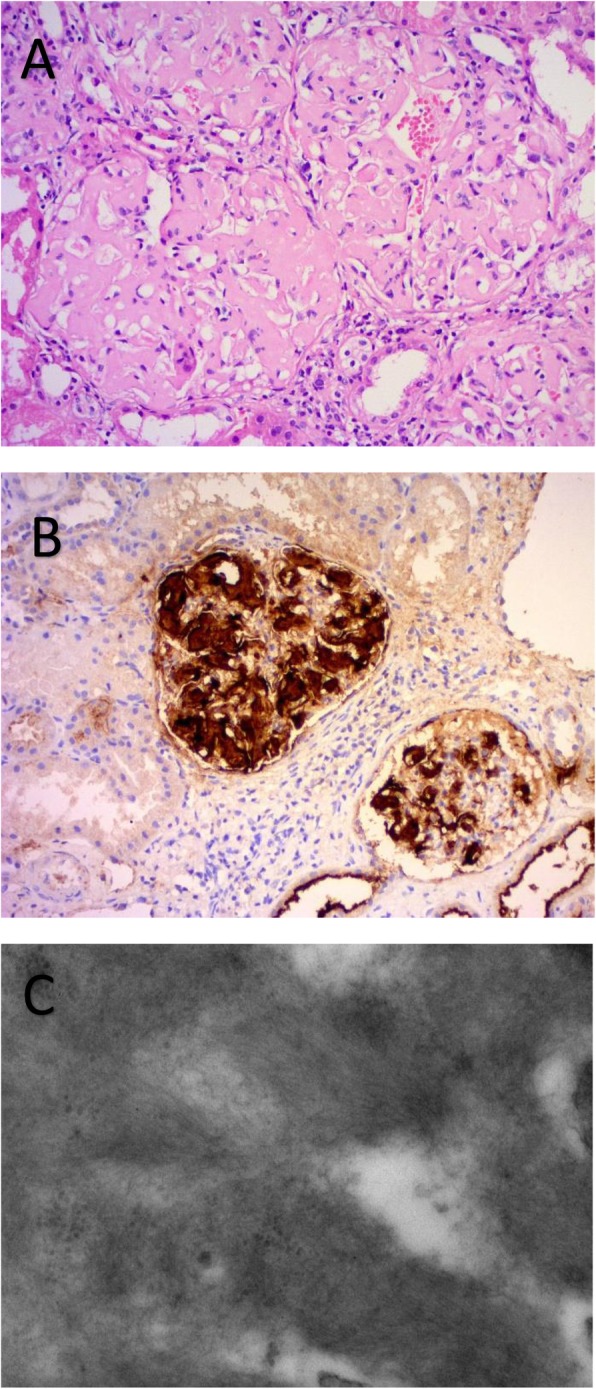
Histology of renal amyloidosis. **a** H&E stain shows extensive effacement of the glomerular architecture by amorphous amyloid. **b** The involvement of glomeruli by AA amyloid are revealed by immunohistochemistry using antibody specific for amyloid A protein. **c** Electron micrograph shows random alignment of amyloid fibrils in the subepithelial zone of a glomerular capillary

For light microscopy, serial two-to-three micron sections were stained with hematoxylin and eosin and other special stains, including Congo Red (Fig. 1).

For all this period, immunohistochemistry for AA amyloidosis was available and performed routinely. Testing for kappa and lambda light chains was carried out via immunohistochemistry for most of the study period, with a few recent cases employing immunofluorescence.

The diagnosis of amyloidosis was confirmed by a positive Congo red staining examined with apple-green birefringence under polarized light. Typical electron microscopic features of randomly disposed, rigid, nonbranching and variably long fibrils were also considered diagnostic.

Case histories were obtained from hospital records and supplemented by relevant laboratory databases. All the diagnoses listed in clinical notes, evidence of treatment of a specific condition (e.g. TB treatment) or pathological confirmation of a disease prior to, as well as 6 months after the date of biopsy findings of amyloidosis, were recorded. Data were analysed and attempts made to determine the type of amyloidosis present if not explicitly stated in the clinical record. This was done considering the clinical history, immunohistochemistry on biopsy, as well as further testing specifically for plasma cell dyscrasias. If a case was indeterminate, it was documented as such. Survival was determined from medical records, hospital registry data or national mortality records.

Demographic data included were age at renal biopsy, ethnicity and gender; clinical data related to presentation and the indication for biopsy; laboratory data were degree of proteinuria (either by 24-h urine collection or estimated from spot urine protein: creatinine ratio), serum creatinine, serum albumin and serum cholesterol as well as HIV antibody testing using ELISA techniques. When multiple entries for the same variable were found, data were captured with preference given in order to the first set of values obtained 1 month before biopsy (presumably taken on admission), the values recorded on the histology report, and lastly any value found from 1 month pre-biopsy to 6 months post biopsy. Case records of the 46 patients were supplemented with additional laboratory data available for 33 of the cases (72%).

### Statistical considerations and sample size

The sample size was limited by the number of cases identified over the study period. The prevalence of amyloidosis on renal biopsy was calculated by dividing the number of cases of amyloidosis by the total number of adult non-transplant biopsies performed over the study period. IBM SPSS version 23 (IBM Corp. Released 2015. IBM SPSS Statistics for Windows, Version 23.0. Armonk, NY: IBM Corp.) was used to analyse the data. Survival was estimated using Kaplan-Meier analysis. Values are reported as medians with inter-quartile ranges (IQR). Non-parametric Mann Whitney tests were used to compare non-normally distributed continuous variables between the two types of amyloidosis. Pearson’s chi-square or Fisher’s exact tests were used compare categorical variables between the types. A *p* value < 0.05 was considered statistically significant.

## Results

Over the 32-year period, 3329 non-transplant renal biopsies were performed and 48 (1.4%) cases of amyloidosis were identified. One patient with amyloidosis on three separate renal biopsies was only counted once. The prevalence of amyloidosis on renal biopsy over the 32-year period was 1.38 per 100 non-transplant renal biopsies. There was a trend to a decline in the biopsy prevalence of all types of amyloidosis over the 32-year period, mainly due to fewer AA cases noted over this time.

The epidemiology, presenting features, laboratory features and survival outcomes are shown (Table 1). The median age at presentation was 51 years (range 21 to 85) and 24 of the 46 patients (52%) were female. The nephrotic syndrome was the commonest presentation in 39 (85%) patients, followed by renal failure (serum creatinine > 120 μmol/L) in 24 (52%).

**Table 1:** Characteristics of 46 cases of renal amyloidosis

	AA	AL	*p* value	Total
Number of cases, *n* (%)	20 (43)	26 (57)		46 (100)
Age (years) at diagnosis, median (IQR)	42 (31–51)	58 (50–66)	< 0.001	51 (41–62)
Sex, *n* (%)			0.74	
Male	9 (45)	13 (50)		22 (48)
Female	11 (55)	13 (50)		24 (52)
Ethnicity, *n* (%)			0.03	
African	10 (50)	7 (27)		17 (37)
White	0	7 (27)		7 (15)
Mixed race	10 (50)	12 (46)		22 (48)
Presentation, *n* (%)			0.59	
Nephrotic syndrome	8 (40)	14 (54)		22 (48)
Nephrotic syndrome and renal failure (serum creatinine > 120 μmol/L)	9 (45)	8 (31)		17 (37)
Renal failure without nephrotic syndrome	3 (15)	4 (15)		7 (15)
Laboratory Features, median (IQR)				
Serum creatinine (μmol/L)	276 (68.5–882.5)	88.5 (82–244)	0.52	172 (81–385)
Degree of proteinuria (g/day)	9.7 (5.1–17.6)	6.2 (4.8–8.2)	0.19	6.76 (4.9–12.0)
Serum albumin (g/L)	18 (16.0–20)	23.5 (18–31)	0.03	19 (16–29)
Serum total cholesterol (mmol/L))	6.12 (5.14–7.8)	8.80 (6.60–12.17)	0.01	7.45 (5.9–11.6)
HIV Status, *n* (%)			0.15	
Positive	2 (10)	0 (0)		2 (4)
Negative	15 (75)	18 (69)		33 (72)
Unknown	3 (15)	8 (31)		11 (24%)
Outcomes				
Survival after diagnosis (months), median (IQR)	1 (0.75–1.25)	9 (1.5–15)	0.02	2 (1–10)

*Abbreviations*: *AA* Secondary amyloidosis, *AL* Primary amyloidosis, *HIV* Human immunodeficiency virus, *IQR* interquartile range

The cases were classified as AL type (26 cases, 57%) and AA (20 cases, 44%). Screening tests to exclude AL amyloidosis (serum or urine protein electrophoresis) were performed in 45% of those with AA amyloidosis. Bone marrow examination was performed in 24 of the 26 AL cases. AA amyloidosis patients were younger (median age 42 years, IQR 31–51 years) compared to those with AL disease (median age 59 years, IQR 50 to 66 years). None of the patients with AA amyloid were white; by contrast 7 (27%) of AL cases were white.

Immunohistochemistry of the biopsy specimen identified 18 (90%) cases with the final clinico-histopathological diagnosis of AA amyloidosis. The remaining two (10%) cases were presumed to be of the AA type as the immunohistochemical staining was indeterminate; one had active pulmonary tuberculosis whilst the second had infective endocarditis and HIV infection, with no monoclonal peak detected in the urine or serum. Only one patient identified as having AA on immunohistochemistry of the renal specimen was found to have a clinico-histopathological diagnosis of AL after subsequent investigations. 13 (50%) cases with the final clinico-histopathological diagnosis of AL amyloidosis were indeterminate on immunohistochemistry or fluorescence, with 11 cases (42%) being correctly identified. This diagnostic uncertainty was primarily due to high background staining, likely from contaminating serum proteins [23]. The remaining cases all had a detectable monoclonal peak in serum, urine or bone marrow analysis and usually no associated predisposing cause for AA amyloidosis on clinical grounds.

Serum albumin was significantly lower in cases with AA amyloidosis (18 vs. 23.5 g/L*, p = 0.03*) and median serum creatinine as well as magnitude of proteinuria was higher in AA compared to AL amyloidosis (serum creatinine 276 vs. 88 μmol/L, proteinuria 9.7 vs. 6.12 g/24 h) but failed to attain statistical significance.

**Fig. 2:**
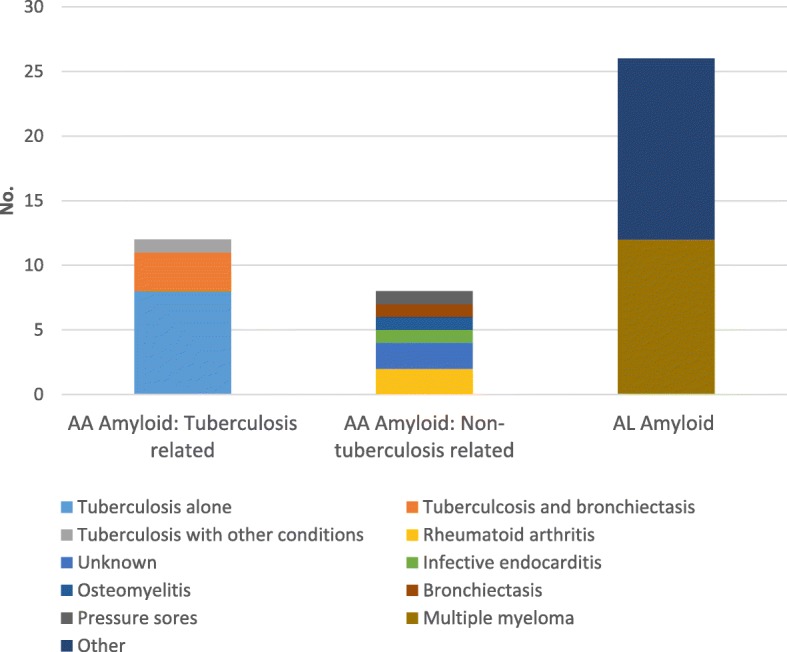
Causes of AA and AL amyloidosis

Of the 20 cases of AA amyloidosis, 12 (60%) were associated with pulmonary TB. Of these 12, three also had bronchiectasis and one patient had active TB as well two other chronic infections (bronchiectasis and bilharzia). Two cases of AA amyloid had no known associations, although both patients had a history of pulmonary TB 10 to 15 years prior to the diagnosis of amyloidosis; the remaining associated conditions in AA amyloidosis are shown (Fig. 2). None of the cases of AA amyloidosis were known intravenous drug users (IVDUs) or had hepatitis B or C infection.

Of the 26 patients with AL amyloid, 12 (42.2%) were diagnosed with multiple myeloma and were treated with steroids, and either melphalan or cyclophosphamide. Of the remaining 14 patients with AL amyloid four received melphalan and steroids; the remainder received no specific therapy. In patients with AA amyloidosis treatment was primarily of the underlying disease as identified. Both HIV-infected patients were anti-retroviral therapy naïve at presentation.

Other organ involvement occurred mostly in patients with AL amyloid: a single patient had amyloid deposits on colonic biopsy and four on the bone marrow biopsy; single patients with AL amyloid had hepatosplenomegaly, macroglossia; and suspicious thyroid lesions on radioisotope scan; two patients with multiple myeloma were diagnosed with carpal tunnel syndrome and three patients had echocardiographic findings suspicious of cardiac involvement. Interestingly, none of the patients with AA amyloid manifested systemic disease at presentation.

HIV status was known in 35 (76%) patients with no test results available before 1992. HIV infection was noted in only two patients (4%); both cases were of the AA type. One of these patients had infective endocarditis and the other had no identifiable association although the patient did have pulmonary TB 14 years prior to biopsy. By comparison, in the total 3329 renal biopsies done over the study period, 481 biopsies were in patients with confirmed HIV, with a total biopsy prevalence for HIV of 14.4%. Analysis of data exclusively in the period after the introduction of HIV testing in 1992 yields a biopsy prevalence of HIV of 5% in the amyloid cohort, and 17% in the overall biopsy cohort.

**Fig. 3:**
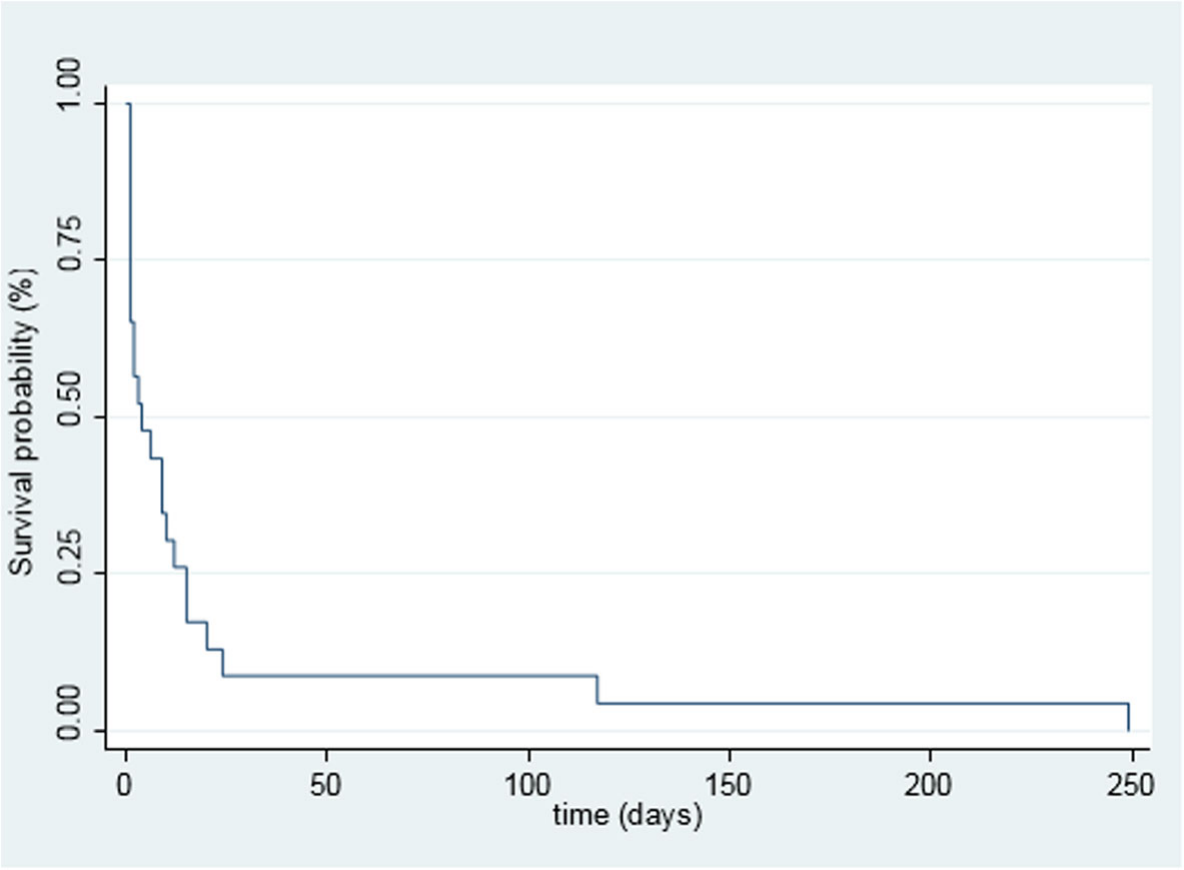
Kaplan-Meier estimate of survival of patients with amyloid (*N* = 25)

Survival data were available for 25 (54%) patients (Fig. 3). The median survival was 2 months after diagnosis with a range of 0 to 249 months. A significantly worse outcome was found in cases of AA amyloidosis, with a median survival of 1 month after diagnosis as compared to 9 months in AL amyloidosis (*p = 0.02*).

## Discussion

Amyloidosis is a rare cause of kidney disease accounting for less than 2 % of all biopsied patients and is most commonly associated with TB in the South African setting. It is most likely to present with nephrotic syndrome. A similar number of AA and AL cases were observed and outcomes were poor. AA amyloidosis patients were noted to be younger compared to AL patients, usually had an identifiable disease associated with amyloidosis established on presentation and were all African or mixed ethnicity. HIV infection in our community appeared not to have a discernible impact on the prevalence of amyloidosis in South Africa in our study.

These observations are consistent with other studies. The high proportion of TB associated cases in AA amyloidosis (60%) was higher than the 40% reported by Mody in 1984. This likely represents the increasing incidence of TB over the past three decades [24].

**Fig. 4:**
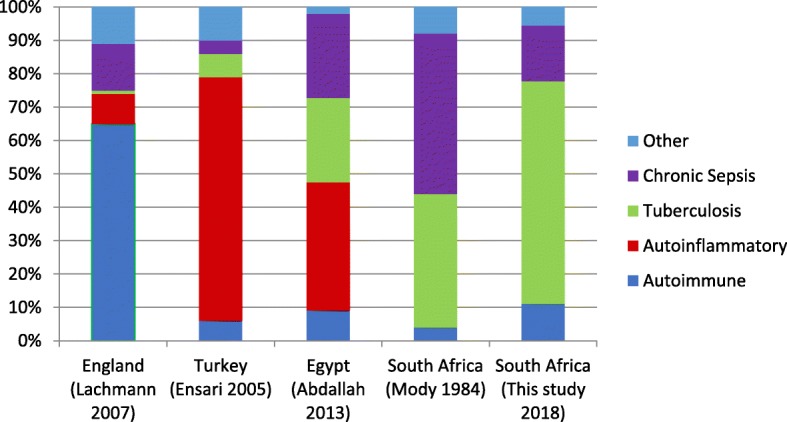
Proportion of diseases associated with AA amyloidosis in different countries [2, 7–9]

The total number of AL cases slightly exceeds those of AA which is unusual for a developing country. This difference was small but difficult to explain, especially considering the high levels of TB noted in South Africa. A comparison of the associated diseases of AA amyloidosis in different regions of the world is shown (Fig. 4).

**Fig. 5:**
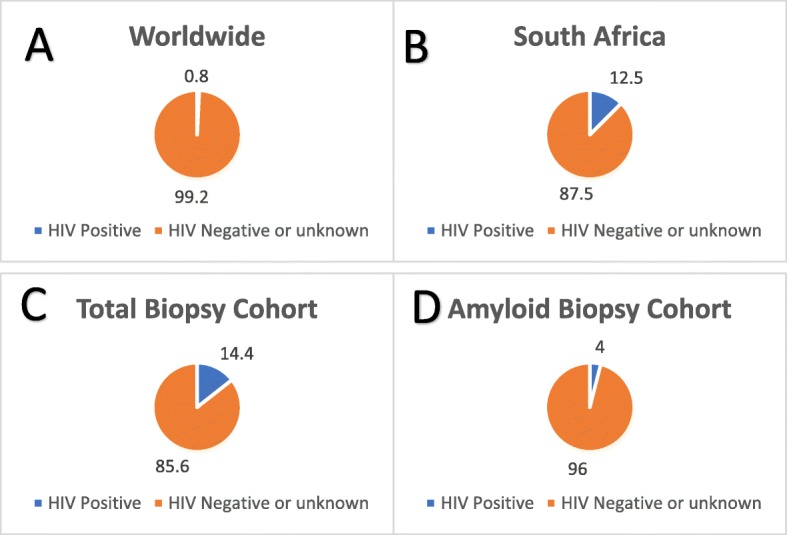
Comparison of HIV prevalence in populations (**a** and **b**) [14, 25] and within our biopsy cohort (**c** and **d**)

Based on the high prevalence of HIV in our setting of 12.6%, we had expected to see many more cases with HIV (Fig. 5). A possible explanation for this is sampling bias. The clinical presentation of HIV associated nephropathy (HIVAN) with nephrotic range proteinuria, renal failure and large kidneys on ultrasound is very similar to that of amyloidosis. However, the standard practice of our institution is to perform a renal biopsy in all cases of suspected HIVAN if not contra-indicated, making this explanation unlikely.

HIV infection has been associated with both AA and AL amyloidosis in case reports [21, 22, 26–28]. Most cases of AA amyloidosis have been reported in IVDUs, fewer cases with visceral leishmaniasis and multicentric Castleman’s disease, and to our knowledge, only 1 case report exists of HIV, amyloidosis and *presumed* TB coexisting in a known IVDU [20–22, 29, 30].

Of the two patients in our study with HIV, one had infective endocarditis which was thought to be directly associated with amyloidosis, whilst the other had no other clinical manifestation apart from HIV infection which could be directly associated with amyloidosis. None of the cases in our series were known IVDUs. Based on a high estimated prevalence of IVDUs in our population [31], this is likely a result of sampling bias in a single centre study.

The association between amyloidosis and HIV is plausible, as higher levels of serum amyloid A (SAA) protein - the acute phase reactant protein from which amyloid fibrils are derived in AA amyloidosis - has been documented in HIV positive individuals [26, 32, 33]. However, substance abuse without HIV infection has also been associated with higher levels of SAA. Samikkannu et al. [28] reported higher levels of SAA in IVDUs, alcoholics and those abusing methamphetamines, independent of underlying HIV infection.

Case series of amyloidosis have revealed discrepant prevalence of HIV in renal amyloidosis. Jung et al. [21] found 12 cases of renal amyloidosis in 24 renal biopsies of IVDUs in a single metropolitan area in Germany, with a significantly higher HIV infection rate (8 cases) in those with amyloidosis than those without amyloidosis (2 cases of HIV). This high percentage of amyloidosis found in this cohort of IVDUs (50% of cases) is much higher than reported in other studies. A Portuguese study of 19 IVDUs, of 3 who also had HIV, reported only a single case of amyloidosis. Similar results were found in an American study of 14 HIV and hepatitis C co-infected IVDUs, where no cases of amyloidosis were found [34, 35].

This discrepancy in the prevalence of HIV in amyloidosis suggests that other confounding factors may be present. Miranda et al. [20] postulate that these discrepancies are largely based on geographical peculiarities and point to skin and soft tissue infection in IVDUs being the driving factor for amyloid deposition, not HIV or hepatitis B or C. Our data, with a low HIV infection rate and no IVDU cases, would support this theory.

Similar to HIV and rather unsurprisingly, TB has also been found to have high levels of SAA [36]. However, we noted no cases of TB (the major predisposition for AA amyloidosis in our study) and HIV co-infection, a finding which is difficult to explain considering the high levels of SAA reported in both conditions together with the 70% HIV/TB co-infection rate found in South Africa. These findings may be corroborated from reviewing the literature, as no case has been described of *definitive TB alone* causing AA amyloidosis in an HIV infected individual.

Thus, the association between amyloidosis and HIV infection is complex and not well understood. Husebekk et al. [37] suggested in 1986 that amyloidosis may not be found in HIV as its course progressed rapidly to demise. However, survival of patients with HIV has improved dramatically since then and this factor alone cannot explain the low frequency of HIV infection amongst our cases. We put forward two novel suggestions about the link between HIV and amyloidosis: firstly that AA amyloidosis may protect an individual from HIV infection as high levels of SAA usually seen in AA amyloidosis have been found to confer some resistance to HIV infection after exposure to the virus [38, 39]; secondly that HIV may even be protective against amyloidosis through impairment of the complex process of fibrillogenesis [40]. Immune dysregulation in HIV, manifesting with alterations in CD4^+^ T helper cell subsets’ function and quantity, may be a mechanism of this impairment of fibrillogenesis [41]. No direct evidence exists to support the second theory and further studies are needed to clarify this association.

The high mortality rate in our patient reflects the advanced state of disease at presentation due to delay in help seeking and/or diagnosis. The exact cause of death was not always known. The survival of a single patient for 249 months in AA amyloidosis due to pulmonary TB suggests that adequate treatment in certain individuals may induce a remission in this disease, but factors related to this could not be identified in our study.

The strengths of this study lie in the extended period over which data were available, the use of various specialised tests to confirm the diagnosis and type of amyloidosis (electron microscopy and immunohistochemistry on biopsy) and the various databases which were searched to obtain and confirm data over this period. The high prevalence of HIV in our setting provided a unique environment to observe the association between HIV and amyloidosis. The weaknesses are primarily related to the nature of retrospective case series which are reliant on completeness of data and observations made at the time of presentation. The inability to test for genetic causes of renal amyloidosis may have led to a misclassification of the type of amyloidosis, although these hereditary causes are uncommon [42]. Due to the small numbers in our study, further statistical analysis to substantiate the impact of HIV on amyloidosis was limited.

## Conclusion

Amyloidosis is a rare cause of kidney disease in our setting with poor outcomes. A similar number of AA and AL cases were observed, and a nephrotic presentation is characteristic. TB is the usual association in AA amyloidosis and highlights its burden on health in South Africa. HIV was not found to have a discernible impact in the prevalence of amyloidosis in South Africa in our study and our hypothesis that HIV infection and amyloidosis may be mutually protective needs further study.

## Data Availability

The datasets used and/or analysed during the current study are available from the corresponding author on reasonable request.

## Abbreviations

AA: Amyloid A or secondary amyloidosis
AL: Light chain or primary amyloidosis
HIV: Human Immunodeficiency Virus
IE: Infective endocarditis
IHC: Immunohistochemistry
IQR: Inter quartile range
IVDU: Intravenous drug user
JIA: Juvenile inflammatory arthritis
RA: Rheumatoid arthritis
TB: Tuberculosis
